# Prevalence of Somatic Symptoms and Somatoform Disorders among a German Adolescent Psychiatric Inpatient Sample

**DOI:** 10.3390/children11030280

**Published:** 2024-02-24

**Authors:** Adam Geremek, Clemens Lindner, Martin Jung, Claudia Calvano, Manuel Munz

**Affiliations:** 1 Psychosomatikum Kiel, 24103 Kiel, Germany; adamgeremek@web.de; 2 Helios Clinic for Child and Adolescent Psychiatry, 24837 Schleswig, Germany; martin.jung@helios-gesundheit.de; 3 Vamed Rehaklinik Damp, Ostseebad Damp, 24351 Damp, Germany; clemens.lindner@vamed-gesundheit.de; 4 Department of Education and Psychology, Clinical Child and Adolescent Psychology and Psychotherapy, Freie Universität Berlin, 14195 Berlin, Germany; claudia.calvano@fu-berlin.de; 5 Clinic for Child and Adolescent Psychiatry, Psychotherapy and Psychosomatics, Center for Integrative Psychiatry, School of Medicine, 24105 Kiel, Germany; 6 Institute for Child- and Adolescent Psychiatry, Center for Integrative Psychiatry, School of Medicine, 24105 Kiel, Germany

**Keywords:** somatoform disorder, child and adolescent psychiatry, screening, early detection, early intervention

## Abstract

Somatoform disorders (SD), commencing during adolescence, represent a major problem in health care systems. While literature underlines the high presence of mental health problems among children and adolescents afflicted by somatic symptoms in the general population, limited evidence is available on the prevalence of comorbid somatic symptoms in child and adolescent psychiatric populations. We assessed the prevalence of somatic symptoms, depression, and anxiety by validated questionnaires in an inpatient cohort. We further screened for the presence of SD. Out of 434 inpatients aged 11–17 years, 371 were included and a total of n = 288 (77.6%) children and adolescents participated in the study. A total of 93.8% of the inpatients reported somatic symptoms within the past six months and still almost half (45.7%) of the sample reported at least one somatic symptom within the last seven days prior to inquiry. Relating to the past six months, 59.5% were positively screened for SD, and 44.6% reported symptoms eligible for positive screening within the past seven days prior to the survey. Somatoform symptomatology was highly associated with anxiety and depression scores, but functional decline was amenable to the number of somatic symptoms only. We provide evidence that somatic symptoms are frequent in children and adolescents being treated in child and adolescent psychiatry and are relevant to everyday functioning. Screening for somatic symptoms should be introduced in the routine diagnostic procedures for early detection of SD in the commencing stages.

## 1. Introduction

Somatoform disorders (SD) are highly chronic and debilitating disorders in adults [1,2]. Unexplained somatic symptoms, at the core of somatoform disorders, most often go unnoticed for a very long time [3]. 

To date, despite the clinical relevance and poor prognosis, only a few studies have reported the prevalence of SD in childhood and adolescence. In a prospective longitudinal study among 3021 adolescents in a community cohort, subthreshold somatoform disorders occurred in 2.7% of the sample [4]. A comparable rate of 3.3% has lately been confirmed in a meta-analysis covering research over the past two decades [5]. Retrospective studies among adults point to the onset of somatoform disorders during adolescence. For instance, an early study [6] uncovered that in 75% (n = 49 out of a sample of n = 65) of patients with somatoform disorders, the first symptoms occurred before the age of 21. Bass and Murphy [7] reported that the first symptoms of adult somatoform disorders present around the age of 14 years. Moreover, it was found that the mean duration of untreated somatoform disorders in their patients was 25 years [3]. This gap between the presumed onset of somatoform disorders in adolescence and later diagnosis in adulthood might be suggestive of a significant delay in diagnosis.

The diagnosis of somatoform disorders according to DSM-IV-TR [8] and ICD-10 [9] has been revised to a great extent in the DSM-5 [10] and ICD-11, which now align with the biopsychosocial approach to a greater extent and presumably facilitate diagnoses. With the introduction of the DSM-5 in 2013 [10], the category of somatoform disorders was substituted by ‘somatic symptom and related disorder’. This changed the clinical focus from a negative perspective of ‘medically unexplained symptoms’ and ‘unexplained somatic symptoms’ and a rather dualistic medical model [1,11,12] into a positive biopsychosocial diagnosis. The diagnosis of ‘somatic symptom disorder’ led to a growing interest in this syndrome in pediatric patients [13,14]. Current aetiological concepts of somatic symptoms integrate both physical and mental processes and their complex interactions, proposing a new classification of ‘functional somatic disorders’ [15]. However, little evidence is available on somatic symptoms or SD in adolescents with mental illness within clinical child and adolescent psychiatric cohorts [16,17].

In general, somatic symptoms account for a significant amount of pediatric outpatient attendances [18,19,20,21]. Such symptoms can occur in any organ system, while abdominal pain, headache, musculoskeletal pain and fatigue are most commonly reported. While the majority of physical sensations are usually self-limiting within a short period of time [21], in some children and adolescents, somatic symptoms persist and may cause substantial impairment in everyday functioning, leading to psychosocial disturbances and, ultimately, to school absenteeism and social separation [22]. Besides biological predispositions and dysfunctional social and environmental circumstances, stress sensitivity and emotional disorders like anxiety and depression were identified as key risk factors for the development and persistence of somatic symptoms [19,21,23]. In line with this, there is an abundance of research on mental disorders in children and adolescents who primarily present with somatic complaints, especially in pain disorders [24,25,26,27,28]; also, on somatic complaints in children and adolescents with emotional complaints in general [29], as well as specific disorders like depression [30], trauma related disease [31], and anxiety disorders [32].

However, only a few studies have focused on somatic symptoms in child and adolescent psychiatric inpatient cohorts who might represent a vulnerable population in the context of somatoform disorders. Livingston and colleagues [33] investigated somatic complaints in a sample of 95 consecutively admitted, psychiatrically hospitalized children. On average, 4.7 somatic symptoms were reported, mostly by children diagnosed with somatoform disorders, psychoses, and separation anxiety. In a mixed outpatient/inpatient clinical sample from a child psychiatric and neuropediatric division, Masi et al. [34] found 69.2% of the patients presented medically unexplained somatic symptoms, with headaches (50.6%) as the most frequent symptom. Screening a Norwegian child and adolescent psychiatry cohort for pain for two years, Mangerud et al. [35] found that 70% among the children and adolescents reported chronic pain, and over a third indicated pain in three or more body locations.

In summary, somatic symptoms are relevant and impairing with respect to everyday functioning [22]. They present quite frequently in general child and adolescent populations [4,5], and are closely intertwined with symptoms of depression [30] and anxiety [32]. Therefore, especially among children and adolescents in mental health care settings, the early identification of somatic symptoms bears the potential to effectively treat and prevent chronic somatoform disorders. However, screening for somatic symptoms and SD oftentimes does not occur in the first place in the German mental health care system.

Therefore, the prevalence and clinical relevance of somatic symptoms and their interrelations with different diagnostic categories and symptoms of depression and anxiety, but also sex and age [30,32], are of particular interest among the highly vulnerable inpatient child and adolescent samples.

In the present study, we investigated the prevalence of somatic symptoms and risk for somatoform disorders in child and adolescent psychiatric inpatients in a major child and adolescent psychiatric clinic during one year. We hypothesized to find substantial portions of somatic symptoms in this clinical cohort even when no SD diagnosis was given. We also assumed to find differences in the prevalence of somatic symptoms and risk for SD in different groups of diagnoses such as affective disorders, emotional disorders, trauma-related disorders, eating disorders, autism spectrum disorders, attention deficit hyperactivity disorder (ADHD), conduct disorders, and substance abuse disorders. Specifically, we expected to find a higher prevalence of somatic symptoms and SD in internalizing disorders such as emotional, affective and trauma-related disorders, and eating disorders as opposed to externalizing disorders such as ADHD and conduct disorders, and diagnoses not categorized as internalizing or externalizing such as substance abuse disorders and developmental disorders. We further hypothesized that somatic symptoms would be correlated with symptoms of anxiety and depression and account for impairment in everyday functioning together with depression and anxiety.

## 2. Materials and Methods

### 2.1. Design and Procedure

Subjects were recruited via the adolescent inpatient departments of the HELIOS Child and Adolescent Psychiatry Clinic in Schleswig, Germany. With 120 child and adolescent inpatient beds, around 1500 admissions per year and a catchment area of around 2 million, the clinic ranges among the largest child and adolescent psychiatric clinics in Germany. In the German health care system, inpatient treatment is offered to children and adolescents with mental health issues experiencing severe impairment in everyday functioning.

**Figure 1:**
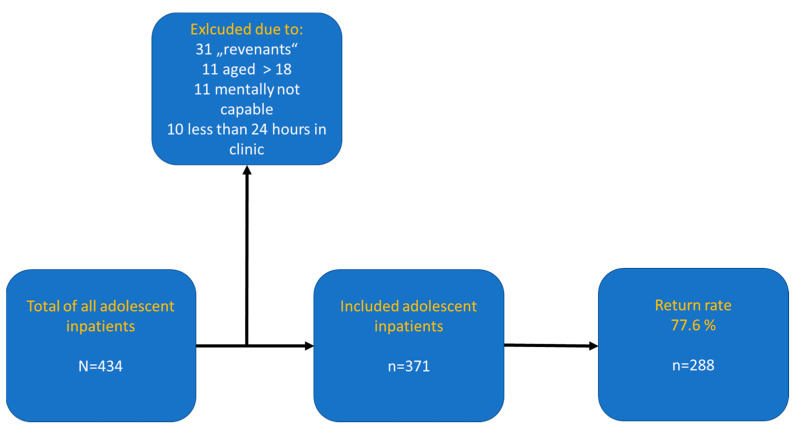
Constitution of the study sample.

All patients were thoroughly assessed by consultants with a specialization in child and adolescent psychiatry, according to ICD-10 [9]. Voluntary participation in the study was offered to all patients being treated during the respective measurement weeks fulfilling inclusion criteria. The study was conducted at four predetermined measurement weeks within one year. To exclude systematic seasonal effects, the seventh week of each quarter (i.e., February, May, August and October) was selected for the assessments. Overall, a total of 434 adolescents between 10 to 18 years were treated in our hospital at these four measurement points. Due to the age range of the validated diagnostic instruments we used, all patients aged >11 were primarily included. One participant was excluded because his parents did not agree to his participation. Exclusion criteria were non-consent of caregivers, severe cognitive or psychopathologic impairment, previous participation, inpatient treatment < 24 h. n = 11 participants were excluded because their age was >18 years. n = 31 participants were also excluded because they had already taken part in the study the quarter(s) before. n = 10 acute psychiatric patients who were at our clinic less than 24 h and n = 11 adolescents who were unable to complete the questionnaires due to cognitive or severe psychiatric impairment (i.e., acute psychotic symptomatology) were also excluded. All patients were informed that participation or nonparticipation would not influence medical treatment in any way. Informed consent was obtained from all participants and their caregivers. Questionnaires were handed out and instructions were given in detail to all participants by the first (A.G.) and second author (C.L.) personally. In this way, the eventual questions of participants were addressed immediately. The study was approved by the local ethics committee and was performed in accordance with the Declaration of Helsinki. Finally, out of the initial 434 adolescent inpatients being treated at the respective study weeks, 371 were enrolled in our study and, among those, 288 fully completed all of the questionnaires and were eligible for the statistical analysis (77.6%, Figure 1).

### 2.2. Measures

#### 2.2.1. Somatoform Symptoms and Somatoform Disorder

The Screening for Somatoform Disorders in Children and Adolescents (SOMS-CA) questionnaire [36] was employed to screen for somatoform disorders. This is a validated self-assessment questionnaire for an age range from 11 to 17 years, which comprises of a list of 31 symptoms (dichotomous yes/no answering format), 17 questions on quality of life and illness-related behavior, and 3 questions covering differential diagnoses (also dichotomous yes/no answering format). The SOMS-CA was developed on the basis of the SOMS adult version [37]. It shows a sensitivity of 97.6%, a specificity of 96.8% for its ability to differentiate healthy children from those suffering from a somatoform condition, and a good internal consistency (α = 0.8) [36].

A total of seven possible points can be scored in the SOMS-CA: one point is awarded for at least one reported somatic symptom, one if no medical explanation for the symptom can be found, one if the wellbeing is impaired by the symptom, one for limitation of the daily routines, one for repeated consultations of physicians, one if the physical soundness cannot be accepted by the patient and one if symptom duration is six months or longer. In order to classify children and adolescents at risk for the diagnosis of a somatoform disorder, the SOMS-screening is positive if participants report at least one somatic symptom and additionally score four of the aforementioned seven possible diagnostic points [36]. To check for current somatoform symptoms, patients were additionally asked whether any of the complaints occurred within the last seven days, since this time span corresponds well to the point prevalence of (somatoform) disorders [38].

#### 2.2.2. Depressive Symptoms

In order to assess depressive symptomatology, the Depression Inventory for Children and Adolescents (DICA) [39] was used. This a validated, well-established, and commonly used German self-report questionnaire to quantitatively measure the grade of depression among children and adolescents. It consists of 26 items (e.g., not liking oneself, loss of interest, etc.) and the symptom presence is reported on a 4-point Likert scale, ranging from 0 (‘no symptom’) to 3 (‘strong symptom’). German normative scores are available, and the results are reported in T-values with T ≥ 60 being pathologic.

#### 2.2.3. Anxiety

We used the anxiety section of the Diagnostic System of Psychiatric Syndromes in Children and Adolescents (DISYPS-CA) [40], which is a validated and widely used German diagnostic tool for the assessment of self-reported anxiety symptoms. Besides a general anxiety score, it includes subscales for social phobia, separation anxiety, and anxiety alertness. It consists of 32 items and the symptoms are reported on a 4-point Likert scale, ranging from 0 (‘not at all’) to 3 (‘very much’). Results are reported in Stanines with T-values.

### 2.3. Data Analysis

Statistical analysis was performed with IBM SPSS Statistics, version 27, for Windows. Descriptive statistics were conducted to assess the prevalence of somatic symptoms among the study sample. Further, the items on quality of life and illness behavior will be descriptively analyzed. The validated cut-off of the SOMS-CA will be applied to categorize the sample in two groups (risk for somatic symptom disorder (SD) vs. no risk). In order to investigate prevalence among different patient groups, patients were categorized following the category of their primary diagnosis (internalizing disorders: affective disorders, anxiety and emotional disorders, adjustment disorders and PTSD; externalizing disorders: ADHD, oppositional defiant disorder/conduct disorder; psychosomatic disorders: eating disorders, somatoform disorders; other categories: developmental disorders, substance disorders; CATEGORY). In order to assess the influence of the diagnosis, age or sex on positive somatoform screening, Chi^2^ with the between factors DIAGNOSIS (affective disorders, anxiety and emotional disorders, adjustment disorders and PTSD, eating disorders, somatoform disorders, developmental disorders, ADHD, conduct disorders, substance disorders), SEX (male, female) and AGE (11–13; 14 and 15, 16 and 17 years) was calculated. With respect to the severity of SD symptomatology by means of SOMS-score, we collapsed the sample into four diagnostic categories: internalizing disorders (comprising affective, anxiety and emotional, as well as trauma-related disorders); externalizing disorders (comprising ADHD and conduct disorders); psychosomatic disorders (comprising eating disorders and somatoform disorders); and disorders of other categories (comprising developmental disorders and substance-related disorders). For frequency analyses of somatic symptoms and positive SOMS-score among diagnostic categories, somatoform and conversion disorders (F44/F45) were excluded, with eating disorders as sole diagnoses in this category. A univariate analysis of co-variance (ANCOVA) with the between factors (diagnostic) CATEGORY (internalizing disorders, externalizing disorders, psychosomatic disorders/eating disorders, disorders of other categories) and SEX (female, male) was calculated, controlling for AGE (covariate). ANCOVA was decomposed with independent sample T-tests. We further conducted Pearson’s correlations of related metric variables to assess associations between SD symptomatology, depression severity, anxiety scores, age, and functional impairment. We further conducted linear regression analyses in order to determine whether functional impairment can be predicted by depression, anxiety, or somatic symptoms.

## 3. Results

### 3.1. Sample Characteristics

Demographic and descriptive statistics of our sample including age, sex, primary diagnoses, depression and anxiety scores, medical examinations with admission and the kind of school are given in Table 1. Indicative of child and adolescent inpatient populations, females were slightly overrepresented (56.0%), and slightly older [females: 15.39 +/− 0.13, males: 14.86 +/− 0.18, F(1,287) = 19.70, *p* = 0.015]. Portions of affective, emotional, and trauma-related disorders, and especially in eating disorders, were higher in females, while males displayed more hyperkinetic, externalizing (conduct disorder), and substance abuse disorders in our sample. Moreover, and in accordance with the distribution of diagnoses with respect to sex, depression [females: T = 68.89 +/− 0.89, males: T = 63.49 +/− 1.14, F(1,287) = 2908.03, *p* < 0.001] and anxiety [females: T = 65.68 +/− 1.14, males: T = 61.02 +/− 0.83, F(1,287) = 1541.47, *p* < 0.001] scores were higher in females compared to males. 

### 3.2. Somatic Symptoms: Frequencies of Single Somatic Symptoms and Number of Symptoms

Since somatic symptoms are characteristic of SD diagnoses, a subgroup of patients with SD diagnosis (n = 10, F44: n = 4, F45: n = 6) was excluded from the frequency analysis. Of the remaining n = 278 inpatients, n = 256 (92.1%) reported at least one somatic symptom during the last six months, and the average number of symptoms was M = 10.16 (SD = 6.43). Prevalence within seven days prior to the survey was n = 127 (45.7%). The most frequent somatic symptoms were fatigue (n = 189, 68.0%), followed by headache (n = 186, 66.9%), abdominal pain (n = 163, 58.6%), vertigo (n = 150, 54.0%), and nausea (n = 149, 53.6%). An overview of the frequency of each single somatic symptom among the 4 symptom categories is given in Table 2.

### 3.3. Somatic Symptoms: Screening for Somatoform Disorders (SD) in Children and Adolescents [35]

In our subsample of n = 278 youths with psychiatric diagnoses except for somatoform disorders, n = 164 (59.0%) participants were positively screened for SD according to the SOMS-CA. In females, the proportion of patients screened positively for SD was higher than in males [females: n = 107 pos/n = 45 neg; males: n = 57 pos/n = 69 neg, Chi^2^(1) = 18.023, *p* < 0.001], and higher in internalizing and eating disorders [internalizing disorders: n = 104 pos/n = 49 neg; externalizing disorders: n = 28 pos/n = 41 neg; eating disorders: n = 17 pos/n = 7 neg; other categories: n = 15 pos/n = 17; Chi^2^(3) = 18.995, *p* < 0.001]. Age in patients screened positively was slightly higher than in those screened negatively [T(df = 268) = −3.371, *p* < 0.001; pos: M = 14.45, SE = 0.12; neg: M = 14.72, SE = 0.19]. Distribution of positive screenings is given in Table 3. Age was positively correlated with the number of complaints (r = 0.203; *p* < 0.001).

In order to analyze the relevance of the diagnostic category and sex on SD severity by means of the SOMS-score, we performed a one-way analysis of variance with the between factors CATEGORY (internalizing disorders, externalizing disorders, eating disorders, other disorders) and SEX (male, female), controlling for age (covariate), which is correlated with the SOMS-score (see 3.4). The ANCOVA revealed a main effect of diagnostic CATEGORY [F(1,3) = 3.250; *p* = 0.022] and also a main effect of SEX [F(1) = 11.481; *p* < 0.001]. However, there was no CATEGORY x SEX interaction [F(1,3) = 0.907; *p* = 0.405].

The ANCOVA was decomposed using post-hoc independent sample T-Tests which revealed that patients diagnosed with an internalizing disorder (M = 4.42; SEM = 2.1) scored higher than patients with an externalizing disorder [M = 2.99; SEM = 2.1; T(220) = 4.79; *p* < 0.001] and patients with diagnoses comprised in ‘other categories’ [M = 3.09; SEM = 1.9; T(183) = 3.348; *p* = 0.001]. Patients diagnosed with an eating disorder (M = 4.50; SEM = 0.31) scored higher than patients with an externalizing disorder [M = 2.99; SEM = 1.9; T(91) = 3.301; *p* < 0.001] and patients with diagnoses comprised in ‘other categories’ [M = 3.09; SEM = 1.9; T(54) = 3.000; *p* = 0.004]. SOMS-score was not different between internalizing disorders and eating disorders (*p* > 0.4) and also not different between externalizing disorders and diagnoses comprised in ‘other categories’ (*p* > 0.8). SOMS-score was also largely higher in females than in males [females: M = 4.48; SEM = 0.15; males: M = 3.24; SEM = 0.20; T(276) = 5.116; *p* < 0.001]. Means and standard errors of means are given in Table 3. Proportions of positive screenings for somatoform disorders were higher in affective disorders, anxiety/emotional disorders, adjustment disorders/PTSD, and eating disorders than proportions of positive screenings in ADHD, ADD/CD, and substance abuse disorders (*p* < 0.02).

### 3.4. Correlations and Multiple Regression Analyses

In order to assess the impact of somatic symptoms on everyday functioning, an impairment score ranging from 0 to 7 was calculated using the quality-of-life items of the SOMS covering several areas of every day functioning (“wellbeing”, “daily routine”, “school”, “leisure time”, “family functioning”, “interpersonal functioning”, “want to visit a doctor”, “consult doctor”). Impairment due to somatic symptoms is correlated with the number of complaints (r = 0.562; *p* < 0.001), depressive symptomatology (r = 0.358; *p* < 0.001), and anxiety score (r = 0.322; *p* < 0.001). Based on these correlations, multiple regression analysis was performed to test if the number of complaints, depressive symptomatology, and anxiety predict impairment through somatic symptoms. The fitted regression model was as follows: SOMS-score = −7.24 + (0.154 × number of complaints) + (0.010 × depression score) + (0.109 × anxiety score). The overall regression was statistically significant [R = 0.605; R^2^ = 0.366; F(3,285) = 52.807; *p* < 0.001]. It was found that only the number of complaints (β = 0.517, *p* < 0.001), but not the depression score (β = 0.084, *p* = 0.172) or anxiety score (β = 0.083, *p* = 0.156), significantly predicted impairment.

For the total sample, somatoform symptomatology by means of SOMS-score was positively correlated with age (r = 0.227; *p* < 0.001), depressive symptomatology (r = 0.341; *p* < 0.001), and anxiety score (r = 0.353; *p* < 0.001). For the subsample of inpatients with internalizing disorders (n = 153), SOMS-score was positively correlated with depressive symptomatology (r = 0.212; *p* = 0.009) and anxiety score (r = 0.288; *p* < 0.001) but not with age (r = 0.073; *p* < 0.3). In the subgroup of patients with externalizing disorders (n = 69), somatoform symptomatology was also positively correlated with depressive symptomatology (r = 0.450; *p* < 0.001) and anxiety score (r = 0.451; *p* < 0.001), but again not with age (r = 0.163; *p* = 0.182). In the subgroup of patients with psychosomatic disorders (n = 34), somatoform symptomatology was positively correlated with age (r = 0.492; *p* = 0.003), but not with depressive symptoms (r = 0.038; *p* = 0.829) or anxiety (r = 0.244; *p* = 0.153). Calculated for the category of other diagnoses (n = 32), somatoform symptoms were positively correlated with depressive symptoms (r = 4.62; *p* = 0.008) but not with age (r = 0.96; *p* = 0.602) or anxiety (r = 2.74; *p* = 0.129).

Based on the above presented correlational analysis, multiple linear regression was used for the entire sample to test if the age, depression score, anxiety score, sex or diagnostic category significantly predicted the SOMS-score. The fitted regression model was as follows: SOMS-score = −1.575 + (0.172 × age) + (0.025 × depression score) + (0.255 × anxiety score) − (0.818 × sex) − (0.178 × diagnostic category). The overall regression was statistically significant [R = 0.476; R^2^ = 0.226; F(5,283) = 59.087; *p* < 0.001]. It was found that age (β = 0.148, *p* = 0.006), depression score (β = 0.143, *p* = 0.026), anxiety score (β = 0.198, *p* = 0.002) and sex (β = −0.191, *p* = 0.001), but not diagnostic category (β = −0.086, *p* = 0.107), significantly predicted SOMS-score.

Multiple linear regression was also performed for the subgroups of internalizing disorders, externalizing disorders, psychosomatic disorders, and other diagnoses. Results are given in Supplement File S1.

## 4. Discussion

In our study, we found a high prevalence of somatic symptoms in adolescent psychiatric inpatients, as the majority of the adolescents reported at least one unexplained somatic symptom within the past six months and almost half of our cohort had at least one symptom during the last seven days prior to the survey. The most frequent somatic symptoms were fatigue, followed by headache, abdominal pain, vertigo, and nausea. We also found that somatic symptoms and positive screenings for SD increase with age, are more present in females than in males, and overrepresented in internalizing versus externalizing disorders. We also found somatic symptoms to be highly correlated with symptoms of depression and particularly anxiety but, using linear regressions, only the number of somatic symptoms explained everyday impairment in patients with comorbid anxiety and depressive symptoms. Analyzing the internalizing and externalizing subgroups separately, somatic symptoms correlated with anxiety or depression but were not associated with age. Of note, regarding the subgroup of psychosomatic disorders, somatic symptoms correlated with age but not with anxiety or depression. By contrast, in the subgroup of internalizing disorders, somatic symptoms were predicted by the extent of symptoms of anxiety.

Our results both confirm and extend previous findings of high prevalence of somatic symptoms in child and adolescent psychiatric populations, since Masi and colleagues [34] described a high number of somatic symptoms (almost 70%) in a mixed inpatient and outpatient neuropediatric/psychiatric cohort. In children with anxiety disorders, Ginsburg and colleagues [32] found unexplained somatic complaints in 96% of the participants. In this way, somatic complaints are not rare, but rather common epiphenomena among children and adolescents with mental disorders. However, as far as we are aware, our study is the first to determine the prevalence of somatic symptoms in a comprehensive child and adolescent psychiatry inpatient cohort, augmenting evidence of previous findings by allowing for analyses of subgroups and the influence of anxiety scores, depression scores, age, and sex.

In contrast to previous findings, the number of roughly 10 symptoms, as revealed by the SOMS-CA [36], is higher. While the presentation of somatic symptoms in community cohorts was reported to present rather monosymptomatic [20,41], on the contrary, polysymptomatic presentation in psychiatric cohorts, like in our sample, appears to be common. In a mixed psychiatric cohort of a mean age of 9.8 years, 4.7 complaints were found on average [33]; our population with a mean age of 15.1 years presented 10 symptoms on average when those with somatoform disorders were excluded. Interestingly, another study reported an average symptom count of over 14 complaints in a cohort of children with somatoform disorders, exclusively [17]. The few (8%) monosymptomatic cases in his study were all younger children. Likewise, our data are conducive with the idea of a developmental sequence from initially mono/oligo- to polysymptomatic presentation from childhood over adolescence to adulthood [42], suggesting a long-lasting sensitization to minor symptoms over the years that leads to a further increase in the amount of complaints [43]. This is further supported by findings that, in adults, the mean duration of untreated somatoform disorders comprises 25 years [3].

In our study, we found that, in the context of somatic symptoms, only the number of complaints, but not the depression or anxiety score, significantly predicted higher impairment in everyday functioning. Research indicates that the number of physical symptoms is correlated with the duration of the depression episodes [44] and the severity of depression [30], and that children with higher levels of somatic complaints, especially chronic pain, display higher global impairment than those with fewer somatic complaints [22,32,41]. Furthermore, there is a significant relationship between high numbers of somatic symptoms and suicide attempts [44], especially for recurrent pain symptoms [45] in adolescents. In this way, (undetected) somatic symptoms may interfere with, and hamper, psychotherapeutic efforts when not carefully considered during the initial diagnostic assessment. Somatic symptoms tend to persist from childhood to adulthood [3,10,11,46]. Factors predicting persistence are, among many others, duration and the number of somatic symptoms [47]. If somatic symptoms persist and interventions are not provided, they can cause significant disability and lead to an adverse academic and social impact [1,2]. In adolescents with depressive disorders, somatic symptoms predict later hospital-based psychiatric care in a dose-dependent manner [48]. In a follow-up community-based study among 16–17 year-old-adolescents in Sweden, somatic symptoms predicted severe adult mental health disorders 15 years later, and the number of somatic symptoms was linked to the incidence of suicidal attempts, bipolar disorders, psychotic disorders, and recurrent and chronic depression [49].

Children and adolescents with mental disorders tend to have maladaptive cognitions, which cause emotional distress, which in turn may contribute to stress-related somatic symptoms [50]. Hence, mental disorders like depression and anxiety are likely to contribute to the persistence of the somatic symptoms. But are mental disorders like anxiety and depression solely responsible for the development of somatic symptoms? There is evidence that not mental disorders themselves are decisive [46]. In fact, rather illness-related self-concepts of the children and their parents [51], as well as emotion regulation [12], contribute to the persistence of somatic symptoms, as anxiety, depression and somatic symptoms share a common etiological pathway [52]. This suggests that adolescents with somatic symptoms need specialized treatment and follow-up, regardless of comorbid mental disorders, or even because of mental disorders. Such treatment should be applied by therapists familiar with functional somatic symptoms, since the effectiveness of psychological interventions may not only depend on the somatic symptoms themselves, but also on illness severity, comorbid disorders or the sex and age characteristics of the patients [53] and their parents, their fears, (mental) health history, and family functioning [54,55]. Thus, screening for such somatic symptoms in child and adolescent psychiatry patients should be obligatory.

In our cohort, we found a significant amount of adolescent psychiatry inpatients screening positive for SD. We are aware that a positive screening based on a questionnaire does not automatically mean the diagnosis of SD itself, which should be confirmed by a trained clinician. One might discuss the clinical validity of complaints not fulfilling the full diagnostic criteria of somatoform disorders according to DSM-5 or somatoform disorders according to ICD-10. However, previous studies have shown increased rates of health care use and disability in adolescents even with subthreshold syndromes [11]. Patients with somatic complaints mostly present to primary care physicians first, “who manage them with varying degrees of enthusiasm and success” [42]. In this way, such screening is important to prevent the stigmatization within the health care system of the so-called “somatizers” and their resulting discrimination by health-care providers as “mentally ill” somatic pretenders. When child and adolescent psychiatrists and psychotherapists finally are involved, these patients then should be taken seriously and given the appropriate treatment with “avoiding the pit-fall of explaining the somatic symptoms in a reductive psychological context” [54]. While most studies on unexplained physical symptoms in adolescents recommend screening patients with such somatic symptoms for further psychiatric affection, we, in turn, recommend screening all child and adolescent psychiatry inpatients for any somatic symptoms—as already postulated for pain symptoms [56]. We put forward the evidence that patients with internalizing symptomatology, and particularly symptoms of anxiety, should be carefully asked for somatic symptoms. When establishing screening for somatic symptoms, emphasis should be put on prognostic factors like number of complaints, their severity, and duration [57]. For this purpose, the SOMS-CA [36] proved suitable. It takes 5–15 min to fill in and 5 min to evaluate. In case the SOMS-CA turns out positive, the diagnosis should be clinically confirmed, and psychotherapeutic treatment should be implemented additionally to the treatment of the psychiatric condition the patients were initially admitted for. In this way, early diagnosis and treatment of SD may prevent further unnecessary medical interventions and high costs due to health care use [58]. Our data suggest that somatic symptoms in child and adolescent psychiatric patients are so frequent that—starting at the age of 11 and especially in patients with internalizing symptoms—screening for somatic symptoms and SD should be integrated in routine diagnostic procedures.

Several limitations of our study have to be reflected when interpreting the results and clinical implications. First, our sample is not small, but rather selective as only one hospital in one specific region in Northern Germany was included. Second, not all participants eligible could be included, so one might suspect a bias towards adolescents who show a basic motivation to cooperate. A sample of adolescents who are not genuinely seeking help but still need health care services would be particularly interesting with respect to otherwise undetected somatic symptoms. Although the SOMS-CA [36] has high sensitivity and specificity, clinical assessment by trained clinicians is warranted to confirm the diagnosis of somatoform diagnoses according to ICD-10. Likewise, both the DICA [39] and the DISYPS-CA [40] are self-report questionnaires and pathologic values do not necessarily mean a diagnosis of depression or anxiety disorder but give rather subjective indications of depressive and anxious symptomatology. We focused on pure prevalence in the first place and conducted multiple comparison and correlation analyses, finding highly significant sex differences for somatic symptom prevalence and correlations of somatic symptom severity with anxiety and depressions scores. In the case of sex differences for somatic symptoms, results are mainly unchanged after Bonferroni-correction for testing of 32 symptoms (α = 0.05/32 = 0.0016). Also, for the number of 7 primary correlational analyses, all of those would survive a Bonferroni correction (α = 0.05/7 = 0.007). In order to present the whole picture, we did not correct for multiple testing in the first place. On the other hand, our data suggest that rates of somatoform disorders are substantially higher when carefully diagnosed. As a further limitation, more psychopathological and sociodemographic variables beyond age, sex, global anxiety, and depression would have allowed us to better understand intercorrelations of somatic symptoms with mental symptoms and the identification of specific risk populations. Also, traditional beliefs in families with respect to body sensations and symptoms might have further explained the occurrence of somatic symptoms when present in psychiatric patients. Lastly, high rates of somatic symptoms in child and adolescent psychiatric inpatients should be confirmed in larger samples and a broader age range to promote the implementation of screening for somatic symptoms in child and adolescent psychiatric cohorts.

## 5. Conclusions

As somatic symptoms that cannot be attributed to a well-defined physical disease are frequent phenomena in adolescent psychiatric inpatients, effective screening should be implemented in routine children and adolescent psychiatric diagnostic procedures. Larger studies including outpatients also might extend the understanding of somatic symptoms in psychiatric care. Finally, after being diagnosed more often through screening, integrating the treatment of somatic symptoms in psychotherapy bears the potential of improving outcomes.

## Figures and Tables

**Table 1:** Sample characteristics—means and standard error of mean—M(SEM).

	Total	Female	Male	Test Statistic	*p*
				F(1,287), X^2^(1)	
age in years, M(SEM)	15.15 (0.11)	15.39 (0.13)	14.86 (0.18)	19.70	0.015
sex (n)	288	161	127		
primary diagnosis (ICD-10)	n (%)	n (%)	n (%)	X^2^(8) = 66.73	<0.001 ***
affective disorders (F31–F32)	47 (16.3)	28 (17.4)	19 (15.0)		
emotional disorders (F40–F42; F93)	50 (17.4)	35 (21.7)	15 (11.8)		
trauma-related disorders (F43)	56 (19.4)	37 (23.0)	19 (15.0)		
eating disorders (F50)	24 (8.3)	24 (14.9)	0 (0.0)		
autism spectrum disorders (F84)	9 (3.1)	1 (0.6)	8 (6.3)		
ADHD (F90.0 & F98.8)	9 (3.1)	3 (1.8)	6 (4.7)		
conduct disorders (F90.1, F91 & F92)	60 (20.8)	14 (8.7)	46 (36.2)		
somatoform disorders (F44 & F45)	10 (3.5)	9 (5.6)	1 (0.8)		
substance abuse disorders (F10–F19)	23 (8.0)	10 (6.2)	13 (10.2)		
quarter (year)				X^2^(3) = 4.95	0.176
first quarter (February)	84 (29.2)	55 (34.2)	29 (22.8)		
second quarter (May)	64 (22.2)	35 (21.7)	29 (22.8)		
third quarter (August)	68 (23.6)	33 (20.5)	35 (27.6)		
fourth quarter (October)	72 (25.0)	38 (23.6)	34 (26.8)		
depression score/T-value: M(SEM)	67.07 (0.73)	69.89 (0.89)	63.49 (1.14)	F(1,287) = 2908.03	<0.001 ***
anxiety score/T-value: M(SEM)	63.63 (0.49)	65.68 (0.52)	61.02 (0.83)	F(1,287) = 1541.47	<0.001 ***
medical examinations					
physical examination (yes/no): n	283/6	157/4	126/2	X^2^(1) = 0.30	0.585
blood sample (yes/no): n	269/19	151/9	118/10	X^2^(1) = 0.55	0.457
electrocardiogram (ECG) (yes/no): n	85/204	53/1087	32/967	X^2^(1) = 0.30	0.142
magnetic resonance imaging (MRI) (yes/no): n	22 (266)	11/150)	11 (116)	X^2^(1) = 0.34	0.562
school				X^2^(11) = 14.71	0.005 *
elementary school and general (n)	182	96	86		
grammar school (n)	64	48	16		
free schools/Danish schools (n)	10	4	6		
no school (n)	28	12	16		
school for children with special needs (n)	4	1	3		

* *p* < 0.05; *** *p* < 0.001.

**Table 2:** Number of patients reporting symptoms during the last six months divided by sex.

Categories	Symptoms	Total 278 n (%)	Female 152 n (%)	Male 126 n (%)	Chi	*p*
Pain symptoms	headache	186 (66.9)	122 (80.2)	64 (50.8)	27.021	<0.001 ***
	stomach pain	163 (58.6)	109 (71.7)	54 (42.9)	23.647	<0.001 ***
	back pain	147 (52.9)	83 (54.6)	64 (50.8)	0.402	0.548
	joint pain	91 (32.7)	52 (34.2)	31 (24.6)	0.332	0.328
	pain in legs/feet/arms/hands	106 (38.1)	62 (40.8)	44 (34.9)	1.006	0.324
	chest pain	103 (37.1)	64 (42.1)	39 (31.0)	3.674	0.062
	eurache	55 (19.8)	34 (22.4)	21 (16.7)	1.411	0.290
	pain during urination	19 (6.8)	17 (11.2)	2 (1.6)	9.965	0.002 **
	pain in or around the genital area	8 (2.9)	7 (4.6)	1 (0.8)	3.581	0.058
	other pain	22 (7.9)	14 (9.2)	8 (6.3)	0.774	0.504
gastrointestinal	nausea	149 (53.6)	108 (71.1)	41 (32.5)	41.086	<0.001 ***
symptoms	vomiting	51 (18.3)	36 (23.7)	15 (11.9)	6.381	0.012
	loss of appetite	143 (51.4)	99 (65.1)	44 (34.9)	25.172	<0.001 ***
	diarrhoea	52 (18.7)	27 (17.8)	25 (19.8)	0.196	0.658
	constipation	42 (15.1)	28 (18.4)	14 (11.1)	2.870	0.090
cardiorespiratory	lump in one’s throat	75 (27.0)	49 (32.2)	26 (20.6)	4.707	0.030 *
symptoms	coughing	117 (42.1)	63 (41.4)	54 (42.9)	0.056	0.813
	shortness of breath or sensation of suffocation	99 (35.6)	70 (46.1)	29 (23.0)	15.945	<0.001 ***
	heart palpitations/heart flutter	126 (45.3)	77 (50.7)	49 (38.9)	3.851	0.050
	tiredness	189 (64.0)	127 (83.6)	62 (49.2)	37.337	<0.001 ***
pseudeoneurological	paralysis, muscle weakness	52 (18.7)	37 (24.3)	15 (11.9)	7.008	0.008 **
symptoms	numbness, pins and needles	94 (33.8)	62 (40.8)	32 (25.4)	7.294	0.007 **
	muscle twitching	92 (33.1)	55 (36.2)	37 (29.4)	1.447	0.229
	heaviness in arms/legs	67 (24.1)	45 (29.6)	22 (17.5)	5.555	0.018 *
	gait disturbances/difficulties in walking	80 (28.8)	54 (35.5)	26 (20.6)	7.454	0.006 **
	visual impairment/double vision	38 (13.6)	22 (14.5)	10 (7.9)	0.184	0.668
	speech disorders, loss of voice, hoarseness	56 (20.1)	39 (25.7)	17 (13.5)	6.339	0.012 *
	seizures	42 (15.1)	27 (17.8)	15 (11.9)	1.844	0.175
	trembling	107 (38.5)	73 (48.0)	34 (27.0)	12.884	<0.001 ***
	dizziness	150 (54.0)	108 (71.1)	42 (33.3)	39.453	<0.001 ***
	loss of consciousness/fainting	30 (10.8)	22 (14.5)	8 (6.3)	4.724	0.030 *
	hearing difficulties buzzing/ringing in the ears	77 (27.7)	43 (28.3)	34 (27.0)	0.059	0.809
last week	point prevalence	127 (45.7)	88 (57.9)	39 (31.0)	20.153	<0.001 ***

* *p* < 0.05; ** *p* < 0.005; *** *p* < 0.001.

**Table 3:** Distribution of positive screenings and means and standard error of the mean—M(SEM)—of the SOMS score.

	SOMS-CA				SOMS Score	
n	Neg: 114	Pos: 164	X^2^		M	SEM
diagnoses	n (%)	n (%)	18.504	*p* = 0.010 *		
affective disorders	16 (34.0)	31 (66.0)			4.4	0.28
anxiety and emotional disorders	16 (32.0)	34 (68.0)			4.5	0.33
adjustment disorders and PTSD	17 (30.4)	39 (69.6)			4.4	0.26
eating disorders	7 (29.2)	17 (71.8)			4.5	0.31
developmental disorders	5 (55.6)	4 (44.4)			3.2	0.83
ADHD	6 (66.67)	3 (33.3)			2.7	0.58
ODD/CD	35 (58.3)	25 (41.7)			3.0	0.27
substance disorders	12 (52.2)	11 (47.8)			3.0	0.35
Total	117 (40.5)	172 (59.5)			4.0	0.13
diagnostic category			18.105	*p* < 0.001 ***		
internalizing disorders	49 (32.0)	104 (68.0)			4.4	0.17
externalizing disorders	41 (59.4)	28 (40.6)			3.0	0.25
eating disorders	7 (29.2)	17 (71.8)			4.5	0.31
other category	17 (53.1)	15 (46.9)			3.1	0.33
sex			18.023	*p* < 0.001 ***		
female	45 (31.6)	107 (68.4)			4.5	0.15
male	71 (55.5)	57 (44.5)			3.2	0.20
age group			10.024	*p* = 0.007 *		
11–13 years	51 (55.4)	41 (44.6)			3.4	0.22
14 & 15 years	27 (33.3)	54 (66.7)			4.0	0.22
16 & 17 years	38 (35.6)	69 (64.4)			4.3	0.20

* *p* < 0.05; *** *p* < 0.001.

## Data Availability

The Raw Data Set is given in the Supplementary Materials.

## Supplementary Materials

The following supporting information can be downloaded at: https://www.mdpi.com/article/10.3390/children11030280/s1, Supplement File S1: Multiple regression analyses.
